# Feasibility of a Telemedicine Urgent Care Program to Address Patient Complaints on First Contact

**DOI:** 10.1155/2020/8875644

**Published:** 2020-10-28

**Authors:** Amy D. Lu, Myla Junge, Jonathan Garber, Anna K. Abramson, Mary A. Whooley, Janeen E. Smith

**Affiliations:** ^1^San Francisco Veterans Affairs Health Care System, San Francisco, USA; ^2^Department of Medicine, University of California, San Francisco, USA

## Abstract

Many health systems employ nurse telephone advice services to facilitate remote triage of patients to appropriate level of care. However, the effectiveness of these programs to reduce ED and subsequent health care utilization remains to be demonstrated. We describe a novel virtual urgent care program implemented within a Veterans Affairs (VA) health care system that interfaces with a nurse telephone advice line and leverages telemedicine tools to rapidly address and resolve nonemergent conditions. During a 4-month pilot period, 104 unique patients received care through the program, and over 85% of patients achieved timely resolution for their urgent complaints on first contact with the health care system. Demonstrating feasibility for such a program has potential implications for the optimization of remote triage and urgent care services to improve health care utilization and outcomes.

## 1. Introduction

Over the last two decades, emergency departments (EDs) have experienced a dramatic increase in visits for nonemergent conditions [1, 2]. This has led to higher patient costs, increased wait times, and potential delays in care. Nurse telephone advice services have been implemented by many health systems to reduce the burden on EDs but typically rely on computerized triage algorithms that are overly cautious, resulting in referral of patients to the ED for conditions that could be managed in less resource-intensive settings. We describe a novel virtual urgent care program interfacing with a nurse telephone advice line implemented at a Veterans Affairs (VA) health system in Northern California.

## 2. Materials and Methods

### 2.1. Program Description

We describe the results of a quality improvement project involving a convenience sample of Veterans who telephoned a nurse advice line during business hours (Monday to Friday, 8 am to 4 pm) between June 1, 2019, and September 30, 2019, at San Francisco VA Health Care System. For instances where the computerized triage algorithm recommended in-person evaluation within 24 hours, telephone triage nurses would contact an urgent care provider (physician or nurse practitioner) to discuss whether the case could be addressed via an on-demand video or telephone visit. For appropriate nonemergency cases, the triage nurse offered the patient the opportunity to be evaluated immediately through a video or phone visit with an urgent care provider.

### 2.2. Measures and Analyses

We used electronic health records to obtain patient demographic information, patient care assessment needs (CAN) comorbidity score [3], chief complaint, telemedicine modality utilized, and original triage recommendation for time frame and location of care. The primary outcome of interest was the proportion of cases that achieved first contact resolution (FCR) [4] which we defined as appropriate diagnosis or treatment within 24 hours or redirection to a less urgent follow-up appointment outside of the 24-hour window. A secondary outcome of interest was the proportion of cases seen in a VA or community ED within 72 hours following initial triage.

Basic patient demographic and clinical characteristics were compared between cases that achieved FCR with those that did not. Student's *t*-test was applied for continuous variables and the Fisher exact test for categorical variables. We constructed multivariable logistic regression to determine the association of video-based telemedicine visits on FCR and subsequent ED visit. Statistical significance was set at a two-sided *α* = 0.05, and analyses were performed using STATA (version 16.0, StataCorp, College Station, TX).

## 3. Results

During the 4-month period, urgent care clinicians were available on 50 unique weekdays. On those days, an average of 36 ± 8 patient calls per day were triaged by the computerized algorithm to the same day (within 24 hours) in-person evaluation. A total of 104 unique patients were offered and agreed to a virtual urgent care visit: 78 visits were conducted by video and 26 were conducted by telephone. All visits were conducted immediately after the nurse advice call. Ninety-two (88.5%) cases achieved FCR. Patient demographic and clinical characteristics were largely similar between those who achieved FCR and those who did not (Table 1). Visits conducted over video had a 7.6 (95% CI: 1.2–47.3, *p*=0.03) odds of FCR and 0.04 (95% CI: 0.003–0.5) odds of subsequent ED presentation than those conducted over telephone when adjusted for demographic and clinical characteristics. Though a large proportion of cases were initially recommended for the same day care at an outpatient clinic, 20% (21/104) of cases were successfully diverted away from the ED, and only 7.7% (8/104) of cases resulted in a subsequent ED visit. Common reasons for failure to achieve FCR were related to technical difficulties connecting with patient via video, insufficient assessment by telephone alone, and patient preference for in-person care (Table 2).

## 4. Discussion

Adoption of virtual urgent care has risen dramatically over the last decade, but few such programs have been linked with a nurse telephone advice line. Our pilot program demonstrated the feasibility of a seamless transfer of patients from an existing nurse-led telephone triage line to an immediate virtual evaluation by an urgent care provider. In over 85% of cases, the first contact resolution was achieved immediately. Patients in our study were on average older, more rural, and more likely to be male than those in previous studies of direct-to-consumer telemedicine platforms [5, 6], suggesting a relatively high acceptability of video visits in a population previously thought to be limited by the digital divide.

Our study was limited by a modest sample size given the availability of urgent care providers on select days during the pilot period as well as generalizability of the VA population and integrated health system to the private sector. However, the success of such a program to resolve patient concerns immediately via telemedicine has implications for change across various health care systems. In addition to saving time, travel, money, and ED clinic resources, virtual urgent care offers the opportunity to care for potentially contagious patients in isolation and to protect patients with noninfectious conditions from exposure to virulent infections.

## Figures and Tables

**Table 1:** Patient and visit characteristics by achievement of first contact resolution (FCR).

Characteristic	Achieved FCR (*N* = 92)	Did not achieve FCR (*N* = 12)	*p* value
Age, mean (SD)	58.7 (16.8)	59.3 (14.8)	0.92

Sex, %			0.410
Male	78 (84.8)	9 (75)	
Female	14 (15.2)	3 (25)	

Race, %	0.485		
White	65 (70.7)	10 (83.3)	
Non-White^a^	14 (15.1)	0	
Declined/unknown	13 (14.2)	2 (16.7)	

Marital status, %			1.00
Married	34 (37.0)	4 (33.3)	
Not married^b^	58 (63.0)	8 (66.7)	

Rurality, %			0.220
Urban	51 (55.4)	4 (33.3)	
Rural	41 (44.6)	8 (66.7)	

CAN score, mean (SD)	70.5 (23.3)	76.2 (22.2)	0.425

Type of CC, %			0.149
Musculoskeletal	31 (33.7)	6 (50.0)	
Dermatologic	13 (14.1)	2 (16.7)	
Gastrointestinal	12 (13.0)	4 (33.3)	
ENT/respiratory	11 (12.0)	0 (0)	
Genitourinary	9 (9.8)	0 (0)	
Other^c^	16 (17.4)	0 (0)	

Initial triage recommended care within 24 hours, %	76 (82.6)	9 (75)	0.456

Initial triage recommended follow-up location, %			0.756
Emergency department	21 (22.8)	3 (25.0)	
Clinic	66 (71.7)	8 (66.7)	
Other^d^	5 (5.4)	1 (8.3)	

Visit type, %			0.001
Video	74 (80.4)	4 (33.3)	
Telephone	18 (19.6)	8 (66.7)	

Abbreviations: SD, standard deviation; CAN, care assessment needs; CC, chief complaint; ENT, ear, nose, and throat. ^a^Non-White category includes Black, American Indian/American Native, Asian, and Native Hawaiian or other Pacific Islander. ^b^Not married category includes never married, divorced, separated, and widowed. ^c^Other category included neurologic, cardiovascular, ophthalmologic, and constitutional complaints. ^d^Other category includes home or missing information.

**Table 2:** Reasons for failure to achieve first contact resolution.

*Case*	Chief complaint	Reason for FCR failure	Telemedicine modality used	Case outcome
1	Calf pain	Persistent pain and no clinic appointment available	Telephone	Seen at VA ED; diagnosed with plantaris muscle strain; and discharged home
2	Athlete's foot	Patient hung up and was not able to be reached within 24 hours	Telephone	Patient reached >24 hours later and advised
3	Body pain	Patient requested face-to-face clinic appointment	Video	Patient seen in primary care clinic within 72 hours
4	Toe pain	Inability to evaluate toe due to patient-side technical difficulties setting up video visit	Telephone	Planned for podiatry appointment, but none available; so, ultimately seen in VA ED
5	Finger pain	Diagnosed with early paronychia which progressed to need for drainage	Video	Seen in community ED for incision and drainage and discharged home
6	Toenail problem	Unable to arrange expedient follow-up with primary care or podiatry within 72 hours	Video	Prescribed oral antibiotics for likely infected toe wound and seen by primary care nurse within 1 week
7	Constipation	Not assigned to primary care team and thus recommended to present to ED for expedited workup	Telephone	Seen in VA ED, treated for constipation with laxatives, and referred for colonoscopy for new diagnosis of iron deficiency anemia
8	Abdominal cramps	Not assigned to primary care team and no new patient appointment available within 72 hours	Video	Seen in community ED and diagnosed with functional abdominal pain
9	Blood in stool	Unable to be connected by phone with patient	Telephone	Seen in primary care clinic and triaged to self-care by nurse
10	Leg pain	Patient presented to the ED before video visit was able to be arranged	N/A	Seen in VA ED; diagnosed with superficial peroneal nerve compression; and discharged home
11	Abdominal pain and diarrhea	Difficulty with clinical assessment over phone	Telephone	Seen in community ED
12	Toe pain	Unable to evaluate toe by video and high risk for diabetic foot infection	Telephone	Referred to community ED where he was admitted for diabetic foot infection

Abbreviations: FCR, first contact resolution; VA, veterans affairs; ED, emergency department; N/A, not available.

## Data Availability

The data used to support the findings of this study are available from the corresponding author upon request.

## References

[1] Redstone P., Vancura J. L., Barry D., Kutner J. S. (2008). Nonurgent use of the emergency department. *The Journal of Ambulatory Care Management*.

[2] Durand A.-C., Gentile S., Devictor B. (2011). ED patients: how nonurgent are they? Systematic review of the emergency medicine literature. *American Journal of Emergency Medicine*.

[3] Wang L., Porter B., Maynard C. (2013). Predicting risk of hospitalization or death among patients receiving primary care in the Veterans health administration. *Medcare Pharmacy*.

[4] Morgan S. L., Lucatorto M., Sapnas K. G., Cone W., Tepper K., Bouchard M. (2019). Mobilizing clinical contact centers in the Veterans health administration: nurses on the front lines. *Nursing Economics*.

[5] Uscher-Pines L., Mulcahy A., Cowling D., Hunter G., Burns R., Mehrotra A. (2016). Access and quality of care in direct-to-consumer telemedicine. *Telemedicine Journal and E-Health*.

[6] Martinez K. A., Rood M., Jhangiani N. (2018). Patterns of use and correlates of patient satisfaction with a large nationwide direct to consumer telemedicine service. *Journal of General Internal Medicine*.

